# Plasma Interferon-gamma is Associated with Poor Treatment Response in Neovascular Age-Related Macular Degeneration

**DOI:** 10.14336/AD.2024.1585

**Published:** 2025-02-21

**Authors:** Alexander Kai Thomsen, Maria Abildgaard Steffensen, Jenni Martinez Villarruel Hinnerskov, Amalie Thomsen Nielsen, Henrik Vorum, Bent Honoré, Mogens Holst Nissen, Torben Lykke Sørensen

**Affiliations:** ^1^Department of Ophthalmology, Zealand University Hospital, Roskilde, Denmark.; ^2^Department of Clinical Medicine, University of Copenhagen, Copenhagen, Denmark.; ^3^Department of Immunology and Microbiology, University of Copenhagen, Copenhagen, Denmark.; ^4^Department of Clinical Medicine, Aalborg University, Aalborg, Denmark.; ^5^Department of Ophthalmology, Aalborg University Hospital, Aalborg, Denmark.; ^6^Department of Biomedicine, Aarhus University, Aarhus, Denmark.

**Keywords:** age-related macular degeneration, immunosenescence, T cells, cytokines, treatment response, systemic inflammation

## Abstract

Age-related macular degeneration (AMD) is a leading cause of visual impairment and blindness in the elderly. Aging is the most important risk factor for AMD, and the aging immune system seems to be involved in pathogenesis. This study investigates the systemic aging immune profile in relation to AMD stage and treatment response. Treatment-naïve patients with neovascular AMD (nAMD), intermediate AMD and healthy controls were included in this prospective study. Participants were examined for systemic aging immune profiles and compared to AMD stage, as well as initial and one-year treatment response in nAMD patients. Flowcytometry was performed to determine T cell differentiation (naïve, central memory and effector memory) and expression of costimulatory markers (CD27, CD28, CD56). Cytokine assays were performed to measure the concentrations of plasma cytokines IFN-γ, IL-1β, IL-2, IL-6, IL-10, IL-12, IL-17, IL-22, IL-27, TNF-α. Polymorphisms of CFH and ARMS2 genes were compared in nAMD patients. Patients with nAMD had significantly higher proportions of central and effector memory CD8+ T cells compared to controls (both *P* < 0.036). nAMD patients had significantly elevated concentrations of IFN-γ, IL-1β, IL-2, and IL-10 (all *P* < 0.05). nAMD patients with poor initial treatment response had a significantly higher concentration of plasma IFN-γ compared to good responders (*P* =0.026). Patients with nAMD had a more advanced systemic aging immune profile with higher levels of T cell differentiation and plasma cytokines compared to controls. Poor initial response had elevated levels of plasma IFN-γ compared to good responders in nAMD.

## INTRODUCTION

Age-related macular degeneration (AMD), the most common cause of irreversible vision loss and blindness, is a chronic, multifactorial disease with a pathophysiology driven by aging, genetic susceptibility and environmental factors [1-4]. The onset of AMD can vary greatly but will usually occur after age 50, with the incidence increasing exponentially with increasing age [5]. One late stage is the development of exudative neovascularizations secondary to AMD, where the key driver is vascular endothelial growth factor (VEGF). Neovascular AMD (nAMD) is treated with repeated intravitreal anti-VEGF injections, which since its introduction has reduced the incidence of blindness caused by AMD greatly [6]. However, treatment response in nAMD patients can vary considerably with a significant proportion of patients experiencing deterioration of visual function regardless of anti-VEGF treatment, and these patients might benefit from additional treatment acting on an additional target [7]. Preceding late-stage AMD is intermediate AMD (iAMD), which is characterized by macular drusen and pigmentary abnormalities. Patients with iAMD are at increased risk of developing late-stage AMD, but far from all do [8, 9].

The reason for the varying onset of AMD, stage of AMD, and treatment response of nAMD might lie in the interplay of aging, genetics and environmental factors affecting the individual’s biological aging. An important factor in biological aging is aging of the immune system characterized by dysfunction and dysregulation of the innate and adaptive immune system [10]. Aging of the immune system is associated with chronic low-grade systemic inflammation, including a remodeling of the T cell differentiation profile, a central component in adaptive immunity. The number of naïve T cells decreases with age as they differentiate into central memory and effector memory T cells. Hallmarks of aging T cell differentiation include loss of the costimulatory receptors CD27 and CD28 [11, 12], as well as upregulation of cytotoxic activity, evident by the increase of the activation marker CD56 [12-14]. Dysregulation of aging T cells can cause tissue damage and production of proinflammatory cytokines. Cytokines play an essential role in inflammation, by leading to recruitment and activation of cytotoxic and phagocytic leukocytes, as well as activating the complement cascade. Especially interferon (IFN)-γ and interleukin (IL)-1β has potent proinflammatory properties and dysregulation of these cytokines might lead to destruction of healthy tissue [15]. This chronic inflammation might cause a dysregulated response in the retina secondary to local accumulation of cellular damage, which could lead to the degeneration and neovascularization present in AMD [12, 16-20].

Genetic susceptibility plays an important role in AMD and treatment response. Two important genetic variations related to increased AMD risk are the complement factor H (CFH) rs1061170 single nucleotide polymorphism (SNP) and the Age-Related Maculopathy susceptibility 2 (ARMS2) rs10490924 SNP [3, 21-24].

Our group has previously found increased levels of effector memory T cells in patients with iAMD and nAMD [12], and increased proportions of CD27-, CD28- and CD56+ T cells in nAMD patients [19, 25]. Furthermore, our and other groups have shown a shift in systemic cytokine profiles, characterized by increased levels of proinflammatory cytokines in AMD patients compared to healthy controls [16, 18, 20, 26, 27]. Our group has also found associations between increased systemic inflammation and treatment response in nAMD [28].

Based on these previous findings, we investigate the degree of immune system aging in AMD subtypes by comparing the systemic profiles of circulating T cell differentiation and proinflammatory cytokines in nAMD and iAMD patients compared to healthy controls. Furthermore, we study the association between these profiles and treatment response to anti-VEGF in patients with nAMD. This might contribute to the understanding of the pathogenesis and reveal novel treatment targets of AMD.

## MATERIALS AND METHODS

### Study Design and Participants

The Danish Neovascular Age-Related Macular Degeneration and Treatment Response (DANEART) study is a prospective cohort study investigating systemic immune phenotypes of patients with AMD of different subtypes. The study is conducted at the Department of Ophthalmology, Zealand University Hospital, Denmark as a single-center study approved by the Regional Committee of Ethics in Research of the Region of Zealand, Denmark (journal no: SJ-768) and performed in adherence with the Declaration of Helsinki. Verbal and written informed consent were obtained from all participants prior to inclusion.

Details of the design of the DANEART study have been reported previously [28]. In brief, treatment-naïve nAMD patients, iAMD patients and healthy controls were included. Exclusion criteria were age < 60 years, inflammatory, autoimmune, cancer and infectious diseases, use of immunomodulating treatment, active smoking, plasma C-reactive protein (CRP) > 15 mg/L, vision-affecting disorders other than nAMD and iAMD, and previous treatment for nAMD.

All participants were examined at baseline. Patients with nAMD were treated according to Danish national guidelines with a loading dose consisting of three intravitreal anti-VEGF (aflibercept, 2 mg) injections with a month’s interval and continued with an individualized treatment plan following the observe-and-plan regimen [29]. Patients with nAMD were examined at the time of diagnosis (baseline), after loading-dose to determine the initial treatment response, and after one year to determine the maintained treatment response. The examination consisted of best corrected visual acuity, slit-lamp biomicroscopy as well as multimodal imaging with color fundus photography, spectral domain optical coherence tomography (OCT), and OCT angiography.

**Figure 1.**
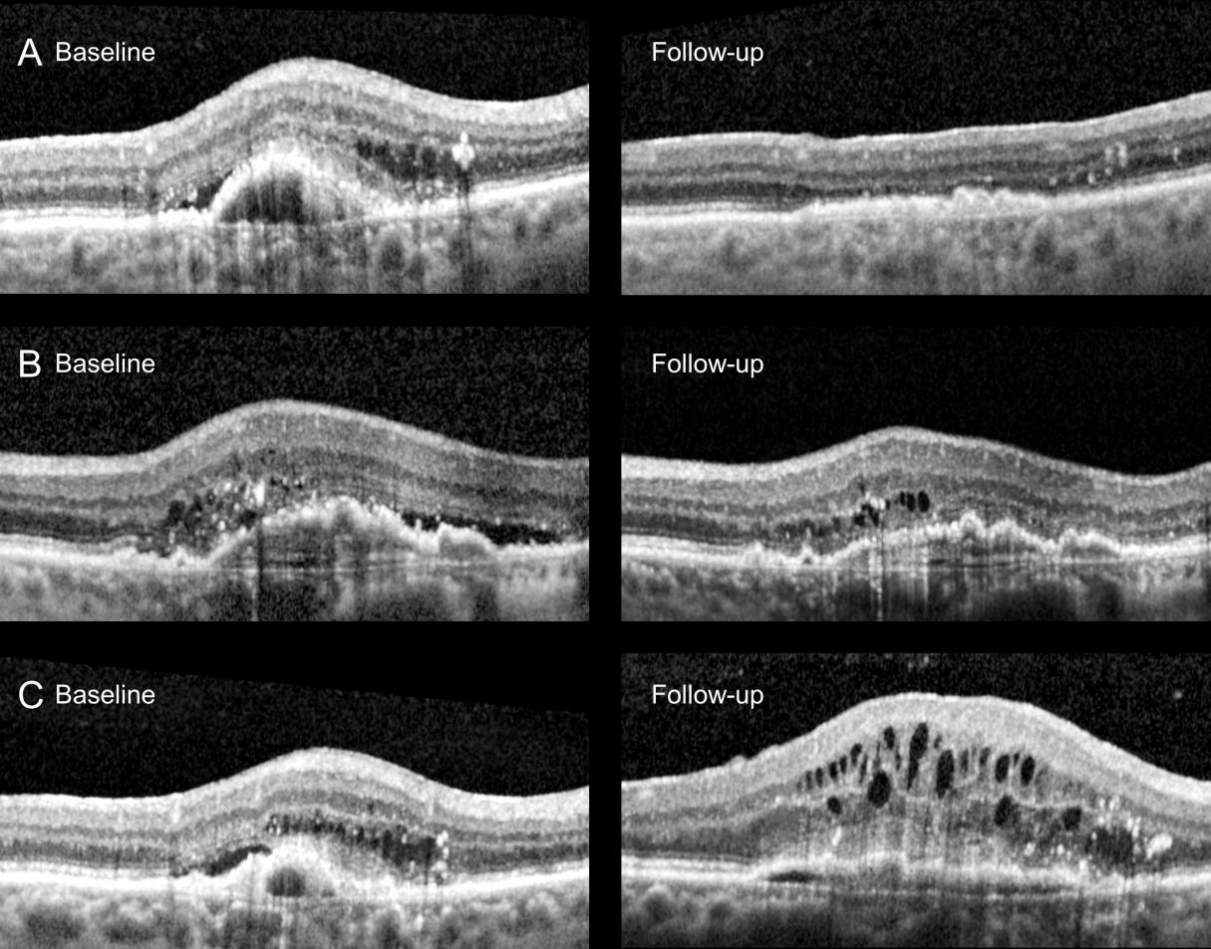
**Examples of treatment response grading in neovascular age-related macular degeneration (nAMD)**. At baseline retinal fluid is present in all nAMD patients as intraretinal cysts or subretinal fluid detaching the neuroretina from the retinal pigment epithelium. (**A**) Good response with absence of retinal fluid at follow-up, (B) Partial response with persistence of retinal fluid and thinning of central retinal thickness (CRT), and (C) poor response, with persistence of retinal fluid and thickening of CRT.

AMD subtype was classified according to the Beckman criteria [30], and treatment response in nAMD patients was graded based on retinal fluid and central retinal thickness (CRT) on OCT. Good responders had total regression of intra- and subretinal fluid, partial responders had persistence of fluid and decreased CRT, and poor responders had persistence of fluid and unchanged or increased CRT [31, 32] (Fig. 1).

### Flow cytometry

**Figure 2.**
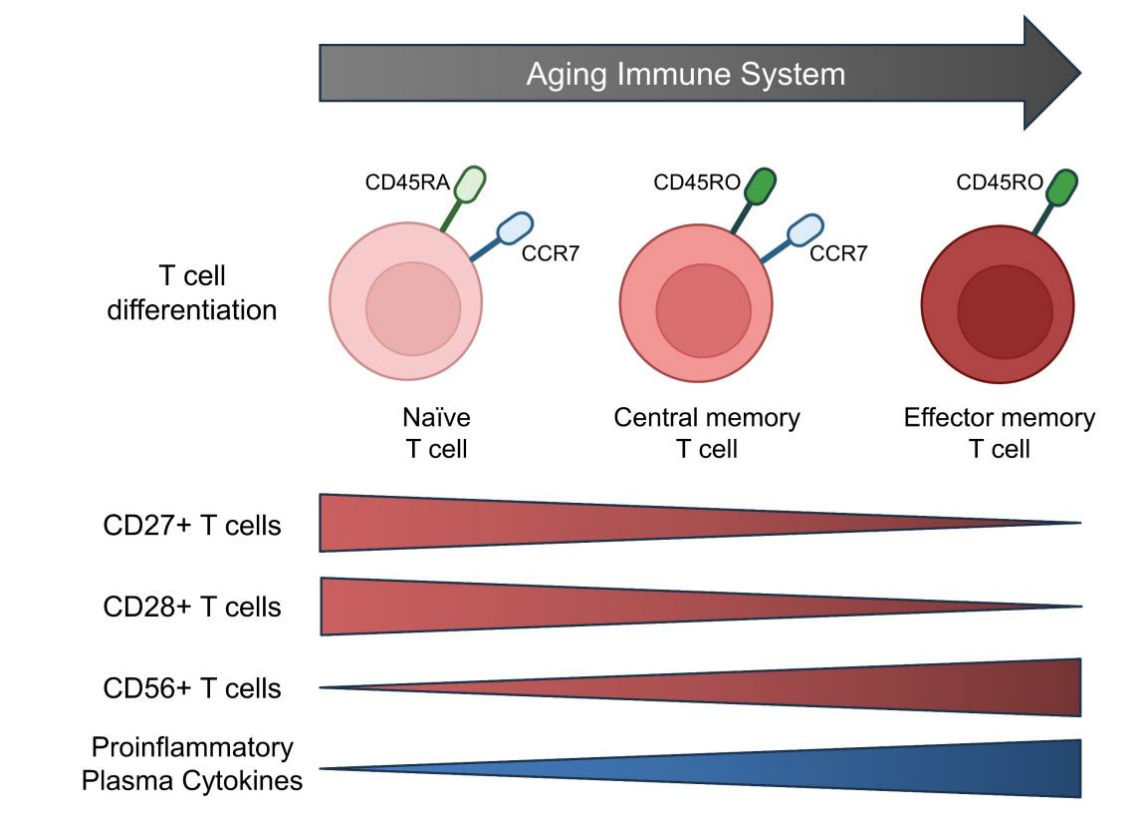
**The aging immune system determined by T cell differentiation, expression of costimulatory markers on T cells, and plasma concentration of proinflammatory cytokines**. An advanced aging profile is characterized by increased levels of central memory and effector memory T cells, lower levels of the costimulatory markers CD27 and CD28, higher levels of the costimulatory marker CD56, and higher levels of proinflammatory cytokines. Made with BioRender.

**Figure 3.**
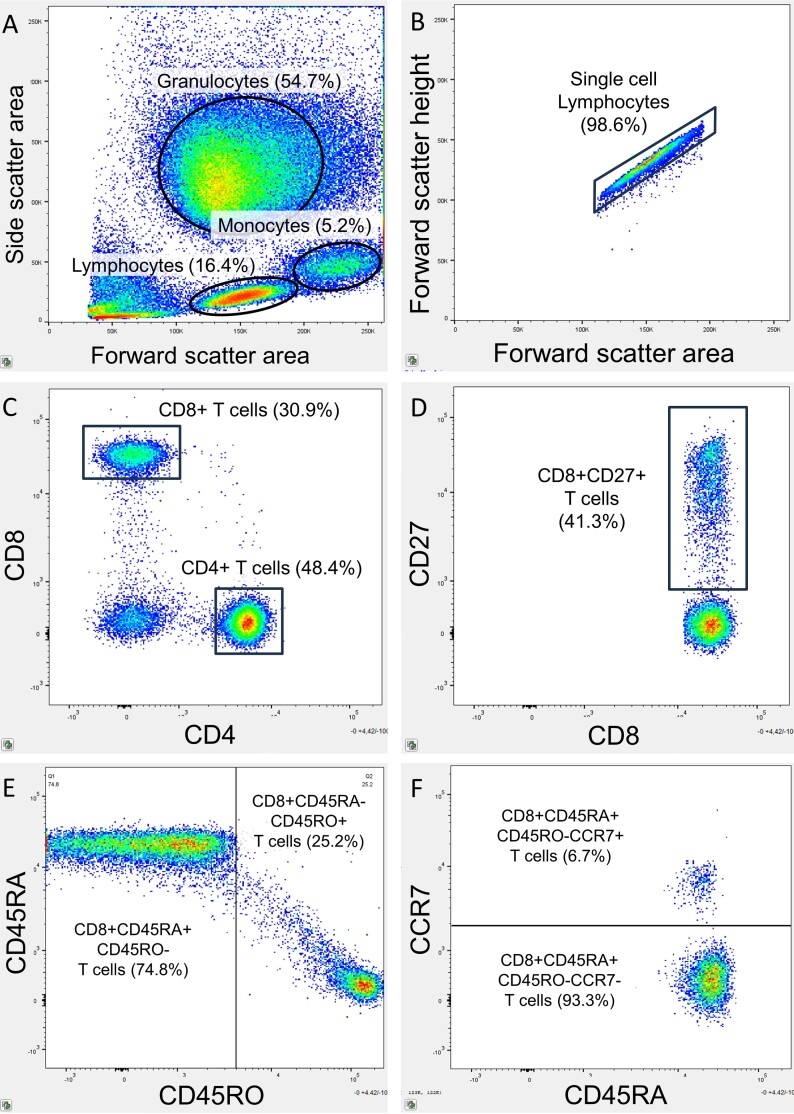
**Flow cytometry gating strategy with Boolean sequences**. (**A**) Lymphocytes were identified on the forward-side-scatter. (**B**) Singlet cell lymphocytes were gated. (**C**) CD4+ T cells and CD8+ T cells were identified among the singlet lymphocytes. (**D**) Co-stimulatory surface markers were identified on T cells, in this example CD27 expression on CD8+ T cells. (**E**) The expression of CD45RA and CD45RO was determined on T cells, in this example on CD8+ T cells. (**F**) The expression of CCR7 was determined on CD45RA+CD45RO- and CD45RA-CD45RO+ T cells, in this example on CD8+CD45RA+ CD45RO- T cells.

To identify the T cell differentiation profile and costimulatory markers at baseline, peripheral blood was sampled to be analyzed with flow cytometry. Blood was sampled from the antecutibal vein in ethylenediamine tetraacetic acid (EDTA) coated tubes and lithium-heparin coated tubes for quantification of plasma CRP concentration. Details of the flow cytometry protocol can be found in Supplementary table 1. Each blood sample was prepared and stained with a panel of monoclonal fluorescent antibodies using fluorescein isothiocyanate (FITC) CD4 (Abcam, cat.no. ab59474), peridinin-chlorophyll-protein (PerCP) CD8 (Biolegend, cat.no. 300922), Brilliant Violet 510 CCR7 (Biolegend, cat.no. 353232), Pacific Blue CD45RA (Biolegend, cat.no. 304123), phycoerythrin-cyanine7 (PE/Cy7) CD45RO (Biolegend, cat.no. 304230), phycoerythrin (PE) CD27 (Biolegend, cat.no. 356406), allophycocyanin (APC) CD28 (Biolegend, cat.no. 302912), and allophycocyanin-cyanine 7 (APC-Cy7) CD56 (Biolegend, cat.no. 300926). Flow cytometry was performed on the BD FACS Canto II flow cytometer (BD Bioscience, San Jose, CA, USA) with a gating size of 100,000 singlet cells. FlowJo analytical software (Tree Star, Ashland, OR, USA, v.10.10.0) was used for the flow cytometric analyses. Gating strategy consisted of identifying lymphocytes on a forward-side scatter plot, followed by gating singlet cells identified on a forward area-forward height scatter plot. Lymphocytes were gated for CD4 and CD8 to identify CD4+ and CD8+ T cells, respectively. T cells were gated for CD45RA, CD45RO and CCR7 to classify the T cell differentiation profile. T cells were classified as naïve (CD45RA+ CD45RO-CCR7+), central memory (CD45RA-CD45RO+CCR7+), and effector memory (CD45RA-CD45RO+CCR7-) [11, 12, 19, 33]. T cells were also gated for the costimulatory markers CD27, CD28 and CD56 (Fig. 2). An example of the gating strategy can be found in Figure 3.

### Cytokine assays

The plasma concentrations of cytokines were quantified with immunoassays. Immediately after phlebotomy, the EDTA coated tubes for the cytokine assays were centrifuged at 1500 × g for 15 minutes. The supernatant was isolated and frozen at -80°C within 1 hour. The cytokine assays were performed on a different day using the commercially available electrochemiluminescence ultrasensitive immunoassay S-plex (Proinflammatory Panel 1 human, cat.no. K15396S-1) analyzing for IFN-γ, IL-1β, IL-2, IL-6, IL-10, IL-12, IL-17 and tumor necrosis factor (TNF)-α, and V-plex (TH17 Panel 1 human, cat.no. K15085D-1) analyzing for IL-22 and IL-27 (Mesoscale Discovery, Rockville, MD, USA). Plasma samples were analyzed in duplicate using the standardized protocol according to manufacturer guidelines.

### Genotyping

Details on genotyping have been reported previously [28]. In brief, the single nucleotide polymorphisms CFH rs1061170 and ARMS2 rs10490924 genotyping was performed on EDTA full blood in patients with nAMD. The blood was frozen at -80°C immediately after phlebotomy and analyzed a different day by BioXpedia, Denmark using the Fluidigm GT192.24 Dynamic Array Integrated Fluidic Circuit (Fluidigm Corp. San Fransisco, CA, USA) according to manufacturer guidelines.

### Statistics

Statistical analysis was performed with R software version 4.2.3 (R Foundation for Statistical Computing, Vienna, Austria). Group comparisons were analyzed with analysis of covariance (ANCOVA) adjusted for age and smoking status (never or previous smoker) between AMD subtypes and nAMD treatment response groups. A logarithmic transformation was applied on the T cell differentiation profiles and plasma cytokines to normalize distributions. Normality was tested with histograms and the Shapiro-Wilk test. In the analysis of AMD subtypes, healthy controls were chosen as reference group. In the analysis of nAMD treatment response, good responders were chosen as reference group. Results of the ANCOVA are presented as mean difference and 95% confidence interval (CI95%) in percentages. Associations between genotypes and T cell differentiation, costimulatory markers and cytokines were analyzed with Wilcoxon rank sum test and presented as median and interquartile range (IQR) in nAMD patients. *P* values were adjusted for multiple testing using the false discovery rate (FDR) method for each compartment. A *P* value < 0.05 was interpreted as statistically significant.

Sample size calculations of healthy controls, iAMD and nAMD patients were based on previous immunological studies of patients with AMD, with an *α* level of 0.05, power of 80%, and effect size of 20%, resulting in a minimum of 30 in each diagnosis group [12, 19, 20]. A direct power calculation on the treatment response analysis was not possible, as no studies have investigated T cell differentiation and these cytokines in nAMD treatment response. Recruitment of nAMD patients continued until reaching 100 participants.

## RESULTS

In the DANEART study, 100 patients with nAMD, 34 patients with iAMD and 61 healthy controls were included. Healthy controls, iAMD patients and nAMD patients had a mean (SD) age of 73 (7), 75 (8) and 80 (6), respectively. Patients with nAMD were significantly older, which was adjusted for in the group comparisons. There were no significant differences in co-morbidities or smoking status (never or previous smoker). Patients with nAMD responded differently to anti-VEGF treatment and of the 100 nAMD patients, 94 completed the 1-year follow-up. The initial (post-loading dose) treatment response of nAMD patients was distributed as 61 (65%) good, 26 (28%) partial, and 7 (7%) poor responders. The distribution of 1-year treatment response was 50 (53%) good, 33 (36%) partial, and 11 (11%) poor responders. There were no significant differences in age, baseline visual acuity or number of injections between the groups after one year [28].

The median and range of the aging immune system markers in the population can be found in Table 1.

**Table 1:** Median and range of aging immune system markers, including proportions of circulating T cells and concentrations of plasma cytokines in the study population (n = 195).

Aging Immune System Marker	Median (range)
**CD8+ T cell differentiation**	
**CD8+ Naïve [%]**	1.8 (0.1 - 20.5)
**CD8+ Central memory [%]**	12.4 (1.3 - 57.7)
**CD8+ Effector memory [%]**	28.9 (3.7 - 76.9)
**CD8+CD27+ [%]**	58.9 (10.3 - 97.5)
**CD8+CD28+ [%]**	48.7 (6.3 - 95.8)
**CD8+CD56+ [%]**	13.1 (1.0 - 66.0)
**CD4+ T cell differentiation**	
**CD4+ Naïve [%]**	13.4 (2.2 - 33.9)
**CD4+ Central memory [%]**	28.6 (7.1 - 60.9)
**CD4+ Effector memory [%]**	12.2 (4.1 - 61.2)
**CD4+CD27+ [%]**	90.4 (13.7 - 98.8)
**CD4+CD28+ [%]**	98.0 (30.4 - 98.8)
**CD4+CD56+ [%]**	66.5 (41.4 - 92.5)
**Plasma cytokines**	
**IFN-γ [fg/ml]**	844 (195 - 12,594)
**IL-1β [fg/ml]**	150 (15 - 595)
**IL-2 [fg/ml]**	182 (46 - 5133)
**IL-6 [fg/ml]**	3178 (566 - 24,974)
**IL-10 [fg/ml]**	812 (86 - 3955)
**IL-12 [fg/ml]**	913 (255 - 5237)
**IL-17 [fg/ml]**	607 (135 - 7557)
**IL-22 [fg/ml]**	1489 (14 - 3147)
**IL-27 [fg/ml]**	2234 (1625 - 8146)
**TNF-α [fg/ml]**	874 (263 - 12,414)

### Aging Immune System and AMD stage

**Figure 4.**
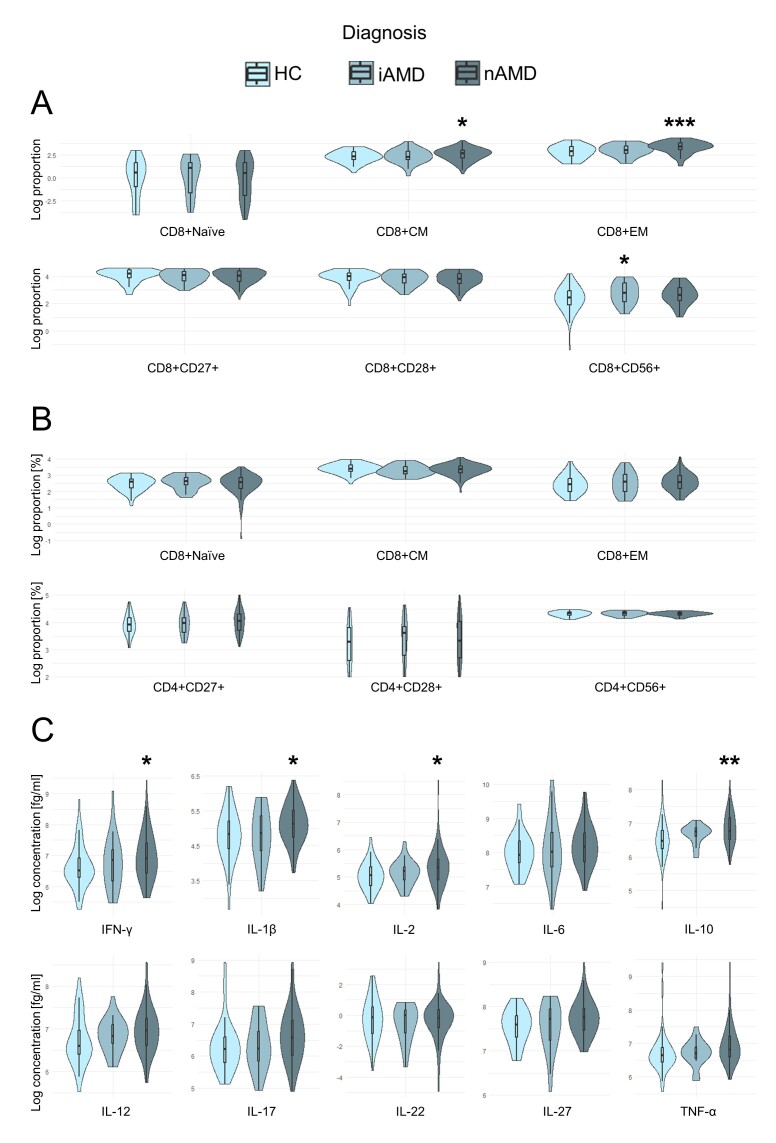
**Violin plots of T cell differentiation, costimulatory markers, and cytokines across AMD stages**. (**A**) logarithm of the proportion of CD8+ T cell differentiation profile and costimulatory markers, (B) logarithm of the proportion of CD4+ T cell differentiation profile and costimulatory markers, (C) logarithm of the concentration of systemic plasma cytokines. The number of healthy controls, iAMD patients and nAMD patients was 61, 34, and 100, respectively. HC = healthy controls; iAMD = intermediate AMD; nAMD = neovascular AMD; CM = central memory; EM = effector memory. * *P* < 0.05; ** *P* < 0.01; *** *P* < 0.001 compared to the reference group (healthy controls) adjusted for age and smoking status with false discovery rate correction.

The T cell differentiation profile differed significantly between patients with nAMD and healthy controls. The proportion of central memory CD8+ T cells was significantly elevated in nAMD patients compared to healthy controls (mean difference: 31%, CI95%: 7%-54%, *P* = 0.036, ANCOVA with FDR correction for 6 tests). Likewise, the proportion of effector memory CD8+ T cells was significantly higher than healthy controls (mean difference: 54%, CI95%: 31%-77%, *P* < 0.001, ANCOVA with FDR correction for 6 tests). The proportion of the costimulatory marker CD56 on CD8+ T cells was significantly increased in patients with iAMD compared to healthy controls (mean difference: 43%, CI95%: 14%-80%, *P* = 0.036, ANCOVA with FDR correction for 6 tests). nAMD patients also tended to have an elevated proportion of CD8+CD56+ T cells compared to healthy controls, however not statistically significant (mean difference: 31%, CI95%: 3%-58%, *P* = 0.064, ANCOVA with FDR correction for 6 tests). Costimulatory markers CD27 and CD28 on CD8+ T cells tended to be lower in AMD patients, but this was not statistically significant (Figure 4A). There were no significant differences in the CD4+ T cell differentiation profile (Fig. 4B).

The cytokine profile was significantly altered in nAMD patients. Patients with nAMD had a significantly elevated plasma concentration compared to healthy controls of IFN-γ (mean difference: 36%, CI95%: 7%-65%, *P* =0.038, ANCOVA with FDR correction for 10 tests), IL-1β (mean difference: 36%, CI95%: 11%-60%, *P* = 0.021), IL-2 (mean difference: 31%, CI95%: 7%-55%, *P* = 0.038), and IL-10 (mean difference: 33%, CI95%: 14%-53%, *P* =0.009). nAMD patients had non-significantly tendencies of elevated IL-12 (mean difference: 20%, CI95%: 1%-40%, *P* = 0.077, ANCOVA with FDR correction for 10 tests), and IL-27 (mean difference: 18%, CI95%: 2%-35%, *P* = 0.055, ANCOVA with FDR correction for 10 tests) compared to healthy controls. No significant differences were found between nAMD patients and health controls of IL-6, IL-17, IL-22, or TNF-α plasma concentrations, or iAMD and healthy controls of any cytokine (Fig. 4C).

### Aging Immune System and Treatment Response in nAMD Patients

**Figure 5.**
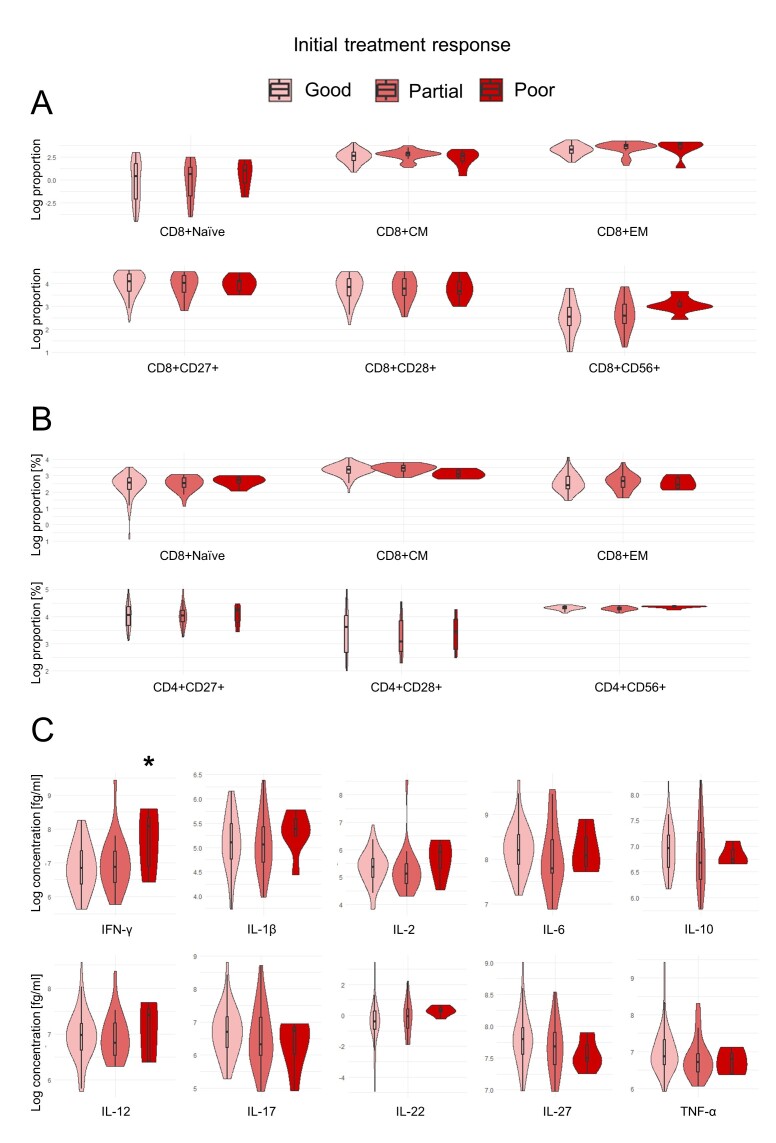
**Violin plots of T cell differentiation, costimulatory markers, and cytokines across initial treatment response of nAMD patients**. (**A**) logarithm of the proportion of CD8+ T cell differentiation profile and costimulatory markers, (B) logarithm of the proportion of CD4+ T cell differentiation profile and costimulatory markers, (C) logarithm of the concentration of systemic plasma cytokines. The number of individuals in the initial treatment response groups categorized as good, partial, and poor was 61, 26, and 7, respectively. CM = central memory; EM = effector memory. * *P* < 0.05; ** *P* < 0.01 compared to the reference group (good responders) adjusted for age and smoking status with false discovery rate correction.

The treatment response to anti-VEGF injections in nAMD patients was evaluated after the loading dose (initial treatment response) and after one year. nAMD patients with a poor initial treatment response had a tendency to have higher proportions of effector memory CD8+ T cells and CD8+CD56+ T cells, however not significant. (Fig. 5A).

**Figure 6.**
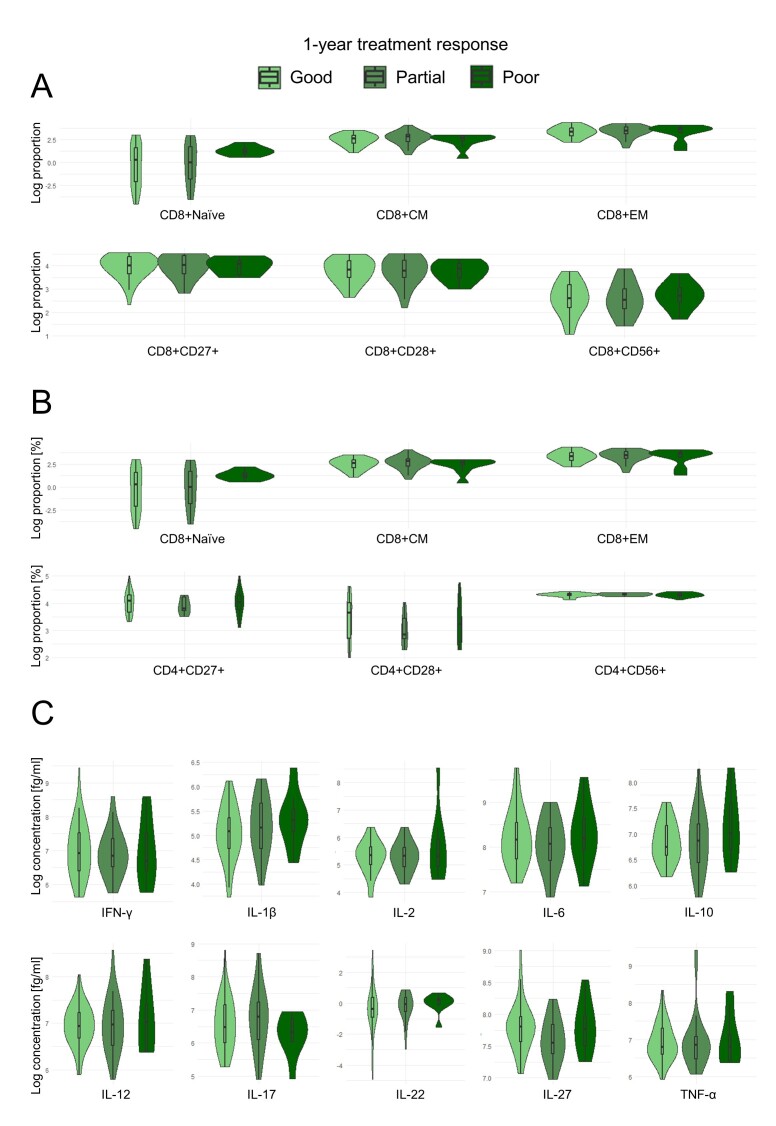
**Violin plots of T cell differentiation, costimulatory markers, and cytokines across 1-year treatment response of nAMD patients**. (**A**) logarithm of the proportion of CD8+ T cell differentiation profile and costimulatory markers, (B) logarithm of the proportion of CD4+ T cell differentiation profile and costimulatory markers, (C) logarithm of the concentration of systemic plasma cytokines. The number of individuals in the 1-year treatment response groups categorized as good, partial, and poor was 50, 33, and 11, respectively. CM = central memory; EM = effector memory. No statistically significant differences were found compared to the reference group (good responders) adjusted for age and smoking status with false discovery rate correction.

Plasma concentrations of IFN-γ were significantly elevated in poor initial responders compared to good initial responders (mean difference: 96%, CI95%: 35%-149%, *P* =0.026, ANCOVA with FDR for 10 tests). Partial initial responders also tended to have increased IFN-γ concentration, however not statistically significant (Fig. 5C).

There were no significant differences between 1-year treatment response groups. There was a non-significant tendency of increased concentrations of IL-2, IL-10, and IL-12 in the poor 1-year responders, and decreased levels of IL-22 in partial and poor 1-year responders compared to good 1-year responders (Fig. 6).

### Aging immune profile charts

To visualize the aging immune profiles of the AMD stages and nAMD treatment response groups, spiderweb charts were created. These charts show the relative differences in expression levels and concentrations for each group, with the highest group value corresponding to 100%, and the other group values as a relative to this magnitude. Spiderweb charts were plotted for the compartments that included statistically significant group differences before adjustments for multiple testing, being the CD8+ T cell compartment and plasma cytokines between AMD stages and nAMD initial treatment response groups.

**Figure 7.**
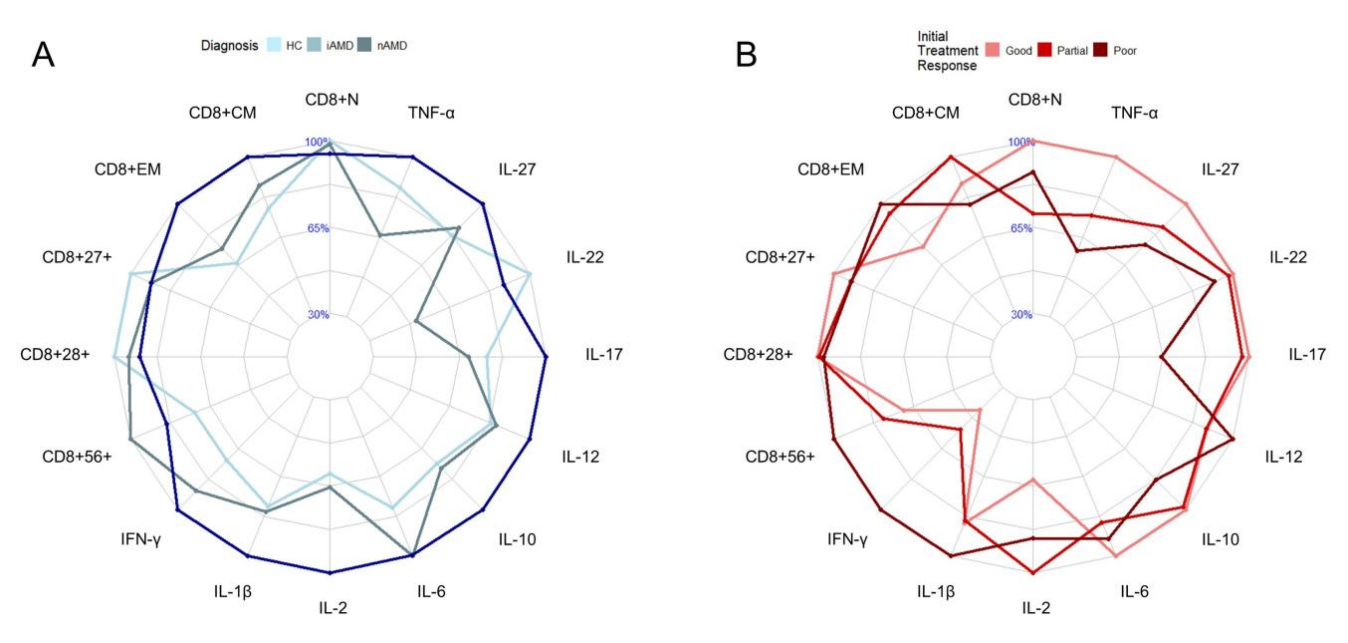
**Aging immune profile charts**. (**A**) AMD stages and (B) initial treatment response groups of neovascular AMD patients. The number of healthy controls, iAMD patients and nAMD patients was 61, 34, and 100, respectively. The number of individuals in the initial treatment response groups categorized as good, partial, and poor was 61, 26, and 7, respectively. HC = healthy controls; iAMD = intermediate AMD; nAMD = neovascular AMD; N = naïve; CM = central memory; EM = effector memory.

Patients with nAMD seemed to have a more advanced profile of the aging immune system compared to healthy controls and iAMD patients. nAMD patients had the highest (most peripheral) level of differentiated CD8+ T cells and most of the plasma cytokines. In addition, this group had the lowest levels of CD27 and CD28 on CD8+ T cells. Patients with iAMD also seemed to have a more advanced aging immune profile compared to healthy controls, but less than nAMD patients. iAMD patients had a higher level of CD56 and differentiated CD8+ T cells, higher level of most of the plasma cytokines, and lower level of CD27 and CD28 compared to healthy controls (Fig. 7A).

The aging immune profiles of initial treatment response in nAMD patients also seemed to differ between groups. The largest relative differences between good and poor initial treatment responders seemed to be poor responders with higher levels of effector memory and CD56+CD8+ T cells, IFN-γ, IL-1β and IL-12, and lower levels of naïve CD8+ T cells, IL-10, IL-17, IL-22, IL-27 and TNF-α. Partial initial responders seemed to have a mix of higher, lower or in between levels compared to the other groups (Fig. 7B).

### Aging Immune Profile and Risk Genotypes in nAMD Patients

Among the 100 included nAMD patients, 82 participants carried the high-risk CFH rs1061170 genotypes (CC or CT), while 18 carried the low-risk genotype (TT). 45 nAMD patients carried the high-risk ARMS2, rs10490924 genotypes (TT or TG), while 55 carried the low-risk genotype (GG). The proportion of CD8+CD28+, CD8+ naïve, and CD8+ central memory T cells were elevated in nAMD patients carrying the high-risk CFH rs1061170 genotypes, however not statistically significant after adjusting for multiple testing (all *P* = 0.062, Wilcoxon rank sum test with FDR correction for 6 tests). No statistically significant differences in plasma cytokines were found across CFH genotypes (Table 2). No significant differences were found in T cell differentiation or plasma cytokines across ARMS2 genotypes (Supplementary Table 2).

**Table 2:** T cell senescence and plasma cytokines stratified according to CFH rs1061170 genotype.

CFH, rs1061170				
	CC/CT (high risk), n = 82	TT (low risk),n = 18	*P* value*	Adjusted *P* value†
**CD8+ T cell differentiation, median (IQR)**				
**CD8+ Naïve T cells [%]**	2.3 (5.3)	0.2 (3.7)	0.029	0.062
**CD8+ Central memory T cells [%]**	15.1 (11.2)	7.9 (12.1)	0.031	0.062
**CD8+ Effector memory T cells [%]**	30.7 (22.0)	28.6 (31.6)	0.87	0.87
**CD8+CD27+ T cells [%]**	58.1 (40.4)	38.4 (36.9)	0.046	0.069
**CD8+CD28+ T cells [%]**	48.2 (33.2)	32.0 (20.2)	0.012	0.062
**CD8+CD56+ T cells [%]**	13.6 (12.4)	14.1 (20.4)	0.42	0.50
**CD4+ T cell differentiation, median (IQR)**				
**CD4+ Naïve T cells [%]**	13.4 (9.0)	12.7 (6.5)	0.83	0.98
**CD4+ Central memory T cells [%]**	28.5 (13.4)	31.9 (11.4)	0.64	0.98
**CD4+ Effector memory T cells [%]**	12.8 (10.6)	12.2 (8.6)	0.67	0.98
**CD4+CD27+ T cells [%]**	94.8 (14.5)	94.7 (19.0)	0.79	0.98
**CD4+CD28+ T cells [%]**	99.6 (10.9)	98.8 (8.1)	0.62	0.98
**CD4+CD56+ T cells [%]**	65.9 (17.1)	66.2 (10.2)	0.98	0.98
**Cytokines, median (IQR)**				
**IFN-γ [fg/ml]**	983 (1189)	1017 (778)	0.63	0.90
**IL-1β [fg/ml]**	172 (139)	160 (90)	0.73	0.90
**IL-2 [fg/ml]**	215 (154)	196 (114)	0.17	0.50
**IL-6 [fg/ml]**	3547 (2715)	3738 (3165)	0.81	0.90
**IL-10 [fg/ml]**	947 (604)	733 (479)	0.19	0.50
**IL-12 [fg/ml]**	1075 (657)	900 (649)	0.25	0.50
**IL-17 [fg/ml]**	651 (651)	1157 (1033)	0.077	0.50
**IL-22 [fg/ml]**	828 (992)	1089 (1208)	0.22	0.50
**IL-27 [fg/ml]**	2323 (975)	2424 (2100)	0.73	0.90
**TNF-α [fg/ml]**	906 (583)	916 (713)	0.97	0.97

* Wilcoxon rank sum test. † *P* values with false discovery rate corrections in each compartment. Bold values indicate statistical significance (*P* < 0.05).

## DISCUSSION

This study aimed to investigate whether the aging immune profiles, characterized as T cell differentiation and plasma cytokine levels, differed between AMD stages compared to healthy controls. An additional aim was to investigate differences in these profiles and treatment response in nAMD patients evaluated after the loading dose (initial response) and after one year.

We found an elevated proportion of differentiated CD8+ T cells in patients with nAMD. These patients had a significantly higher level of T cell differentiation characterized by elevated proportions of central memory and effector memory CD8+ T cells compared to healthy controls. We also found that iAMD patients had a significantly higher proportion of CD8+CD56+ T cells compared to healthy controls, and an overall tendency of advanced T cell differentiation. The concentration of multiple proinflammatory plasma cytokines was also significantly increased in nAMD patients compared to healthy controls. These results suggest that nAMD patients have advanced aging of the immune system and chronic inflammation, which may play a role in the pathogenesis of the disease.

Previous studies have found similar results regarding T cell differentiation in nAMD patients. A study found nAMD patients had a higher proportion of effector memory CD8+ T cells compared to patients with myeloproliferative neoplasms without drusen, a condition with increased systemic inflammation and incidence of AMD [12]. Other studies found the proportion of CD8+CD56+ T cells was increased in nAMD patients compared to healthy controls [19, 25]. The differentiation of CD8+ T cells is especially important, as this compartment is most affected by aging [34]. Memory CD8+ T cells have cytotoxic properties and can cause tissue damage if dysregulated [35] and have been found to be associated with retinal degeneration [36, 37]. A previous study also found an increased proportion of cytotoxic CD8+ T cells in eyes with drusen [38].

Another important function of memory CD8+ T cells is secretion of proinflammatory cytokines [39]. Increased concentrations of the proinflammatory cytokines IFN-γ, IL-1β, and IL-2, as well as anti-inflammatory cytokine IL-10 was found in nAMD patients in our study, and might be important in nAMD development [16-18]. Previous studies also find nAMD patients to have increased levels of systemic IFN-γ [40], IL-1β [20] and IL-10 [20, 26], while other studies find no difference or decreased levels of IL-2 [41]. The increase in the proinflammatory cytokines might be involved in the pathogenesis of nAMD as systemic IFN-γ promotes a shift towards the proinflammatory M1 macrophages [15], IL-1β stimulates retinal inflammation and neovascularization upon entrance to the choroidal vessels following activation by inflammasomes [42], and IL-2 is involved in the migration of retinal pigment epithelial cells and synthesis of extracellular material [44]. The increased levels of the anti-inflammatory cytokine IL-10, might be explained by the age-related increase [43, 44] which stimulates a shift towards pro-angiogenic macrophages [45], and might thus be a rescue mechanism to obtain homeostasis in the increased proinflammatory milieu [46]. We did not find a significant difference between AMD patients and healthy controls in IL-6 or TNF-α plasma concentrations, that has been previously reported [20, 26, 47, 48]. This disagreement may be caused by differing methods of performing cytokine assays or the inclusion of current smokers. We find that iAMD patients tended to have a more advanced aging immune profile, however not statistically significant. We speculate that this observation may result from iAMD patients being a heterogeneous group of patients who will develop nAMD later in life and those who remain with iAMD. Thus, the iAMD patients with a similar profile to the nAMD patients may be more likely to develop nAMD, whereas iAMD patients who do not progress may possess a distinct profile more closely resembling that of healthy controls.

The CFH and ARMS2 genotypes are strongly associated with development of AMD and has been shown to be associated with increased activation of the complement cascade systemically [21, 49, 50]. However, we found no correlations between the CFH rs1061170 or ARMS2 rs10490924 SNPs and T cell differentiation or plasma cytokine levels in nAMD patients. This suggests that the risk genotypes of these SNPs are unlikely to contribute to the systemic changes observed in this study. These findings underscore the multifactorial nature of AMD, where multiple pathways interplay in the disease's development.

To our knowledge, this is the first study to investigate the systemic levels of T cell differentiation and proinflammatory cytokines according to treatment response in nAMD patients. The treatment response was grouped in good, partial and poor assessed post-loading dose (initial response) and after one year. We find that nAMD with a poor initial response had a significantly higher plasma concentration of IFN-γ compared to good initial responders. The diminished therapeutic response to anti-VEGF might especially lie in the effects of IFN-γ. This cytokine has potent proinflammatory properties, causing an inflammatory stress reaction promoting angiogenesis [51]. This might create a retinal angiogenic environment, promoting and maintaining the neovascularization in poor treatment responders, that anti-VEGF might not be able to counter [52]. We do not find any statistically significant differences between the aging immune profile and 1-year treatment response groups. This is likely due to the great variability after one year, where an individualized treatment plan is followed, opposed to the identical treatment protocol of the loading dose [53]. There might thus be conflicting factors that obscure the effects of the aging immune profiles.

Chronic, low-grade inflammation that develops with aging is a hallmark of the aging immune system often referred to as inflammaging. Inflammaging is a consequence of biological aging, resulting from chronological aging, genetics and environmental factors [54]. These are the very risk factors for AMD, and our results suggest that inflammaging might be the reason why some people develop nAMD [16, 17], and why some nAMD patients respond poorly to treatment. Systemic inflammaging can manifest and cause tissue damage in several organ systems [55-57] and is associated with multiple age-related diseases, such as Alzheimer’s disease, cardiovascular diseases, diabetes and cancer [58-62]. Likewise, systemic inflammatory changes of multiple pathways have been identified in AMD patients. This includes increased activation of the complement system [28, 49, 50], T helper cells [63], monocyte activation [64-66], CRP [24, 67], chemokines and chemokine receptors [68-70], as well as systemic inflammation in mouse models [71-73]. The pathways of the immune system interact to a great extent [16, 74] and our findings of an advanced aging immune profile fits in the larger picture of the pathophysiology caused by inflammaging. The advancement of the systemic aging of the immune system might cause the retinal damage, by increasing the proinflammatory milieu locally in the retina [4], or as a two-level model, where age-related stochastic accumulation of molecular damage in the retina combined with the proinflammatory host response causes AMD [16]. In cultured RPE cells, CD56+ T cells were shown to secrete IFN-γ, which induced the production of VEGF from the RPE cells [75]. In another RPE cell culture study, IFN-γ and IL-1β induced reactive oxygen species in RPE cells, which caused oxidative stress leading to protein, DNA and RNA damage [76]. A human study found an increased VEGF expression in RPE cells in response to IFN-γ and IL-1β [77]. These damages and proangiogenic environment at the RPE barrier causes choroidal endothelial cell migration and proliferation, which can lead to the neovascularizations seen in nAMD [4].

The variability in treatment response to anti-VEGF injections in nAMD patients has been a challenge since the introduction of the treatment [53]. However, numerous measures have been developed to create a personalized treatment, including the individualized planning of injection intervals. These regimens include the treat-and-extend and observe-and-plan regimens, which adjust the intervals depending on the previous response [53, 78]. Personalized medicine continues to drive significant advancements across various fields [79], and the time has also come to nAMD [80]. Patients with poor response might benefit from additional or alternative treatment acting on an alternative target than ocular VEGF. Our results suggest that nAMD patients have a more advanced systemic T cell differentiation and higher plasma cytokines levels. Specifically, poor responders exhibit elevated plasma IFN-γ levels, which could serve as a potential predictor of this response and as a target for novel treatments to be explored in future studies.

This study is limited by its observational design, which prevents drawing definitive conclusions about causality. The categorical classification of treatment response was based on a system designed for clinical implementation [31, 32], however the categorical nature limits the ability to capture nuanced differences. The relatively few patients in the poor response group were also a limitation, as potential significant correlations might be hidden.

In conclusion, the advanced aging of the immune system was present in patients with nAMD and to a lesser extent in iAMD patients compared to healthy controls. Poor initial response in nAMD patients was associated with elevated plasma concentrations of IFN-γ.

## Supplementary Materials

The Supplementary data can be found online at: www.aginganddisease.org/EN/10.14336/AD.2024.1585.
