# Supplementary material for: Plasma Interferon-gamma is Associated with Poor Treatment Response in Neovascular Age-Related Macular Degeneration
Source: Aging Dis. 2025 Feb 21;17(2):1068–83. doi: 10.14336/AD.2024.1585 (PMC12834411; doi:10.14336/AD.2024.1585)
Supplement: Supplementary file 1 — The Supplementary data can be found online at: www.aginganddisease.org/EN/10.14336/AD.2024.1585. [file AD-17-2-1068-s.pdf]

SUPPLEMENTARY DATA

Plasma Interferon-gamma is Associated with Poor Treatment Response in Neovascular Age-Related Macular Degeneration

Alexander Kai Thomsen, Maria Abildgaard Steffensen, Jenni Martinez Villarruel Hinnerskov, Amalie Thomsen Nielsen, Henrik Vorum, Bent Honoré, Mogens Holst Nissen, Torben Lykke Sørensen

© 2025. Thomsen AK et al. Published online at http://www.aginganddisease.org/EN/10.14336/AD.2024.1585

SUPPLEMENTARY DATA

Supplementary Table 1. Flow Cytometry Protocol

Flow cytometry preparations were initiated within 4 hours of phlebotomy.
Leukocyte count was performed on Sysmex KX-21NTM (Sysmex Corporation, Kobe, Japan) to calculate blood volume sufficient to obtain 1.0×10^6 leukocytes for analysis.
1% lysis buffer was added to the blood sample to lyse erythrocytes.
Blood sample was stored at room temperature in the dark for 10 minutes.
Cells were washed by adding BD FACS Flow isotonic buffer to the sample, centrifuging at 500 × g for five minutes, followed by decantation of the supernatant. This step was repeated a total of three times.
The isolated leukocytes were resuspended in isotonic buffer.
Monoclonal fluorescent antibodies were added.
The sample was incubated for at room temperature in the dark for 20 minutes.
The stained leukocytes were washed and resuspended in isotonic buffer a last time.
The sample was analyzed on the BD FACS Canto II flow cytometer (BD Bioscience, San Jose, CA, USA) with a gating size of 100.000 singlet cells analyzed per sample.

© 2025. Thomsen AK et al. Published online at http://www.aginganddisease.org/EN/10.14336/AD.2024.1585

SUPPLEMENTARY DATA

Supplementary Table 2. T cell senescence and plasma cytokines stratified according to ARMS2 rs10490924 genotype.

	ARMS2, rs10490924			
	**TT/TG (high risk), n = 45**	**GG (low risk), n = 55**	**P value***	**Adjusted P value†**
CD8+ T cell differentiation, median (IQR)				
CD8+ Naïve T cells [%]	2.4 (4.9)	1.6 (7.5)	0.24	0.49
CD8+ Central memory T cells [%]	15.1 (10.1)	13.6 (14.1)	0.80	0.94
CD8+ Effector memory T cells [%]	27.9 (25.1)	34.2 (14.0)	0.15	0.49
CD8+CD27+ T cells [%]	66.9 (38.0)	54.9 (43.4)	0.32	0.49
CD8+CD28+ T cells [%]	53.5 (30.4)	41.3 (40.2)	0.33	0.49
CD8+CD56+ T cells [%]	13.4 (12.9)	14.1 (14.4)	0.94	0.94
CD4+ T cell differentiation, median (IQR)				
CD4+ Naïve T cells [%]	13.6 (6.7)	12.6 (11.7)	0.76	0.76
CD4+ Central memory T cells [%]	27.8 (13.5)	30.4 (11.1)	0.13	0.76
CD4+ Effector memory T cells [%]	11.2 (10.1)	13.6 (11.2)	0.51	0.76
CD4+CD27+ T cells [%]	88.3 (13.0)	88.9 (20.2)	0.74	0.76
CD4+CD28+ T cells [%]	98.0 (8.6)	97.8 (17.0)	0.42	0.76
CD4+CD56+ T cells [%]	66.0 (14.1)	65.5 (17.2)	0.69	0.76
Cytokines, median (IQR)				
IFN-γ [fg/ml]	810 (1228)	1002 (909)	0.43	0.90
IL-1β [fg/ml]	161 (116)	170 (151)	0.69	0.90
IL-2 [fg/ml]	214 (161)	204 (122)	0.73	0.90
IL-6 [fg/ml]	3837 (3163)	3143 (2418)	0.37	0.90
IL-10 [fg/ml]	830 (698)	1008 (577)	0.23	0.90
IL-12 [fg/ml]	1046 (717)	1061 (603)	0.70	0.90
IL-17 [fg/ml]	649 (774)	796 (799)	0.63	0.90
IL-22 [fg/ml]	814 (1081)	916 (896)	0.90	0.90
IL-27 [fg/ml]	2380 (1028)	2323 (1389)	0.87	0.90
TNF-α [fg/ml]	983 (487)	897 (788)	0.41	0.90

* Wilcoxon rank sum test.
† *P* values with false discovery rate corrections in each compartment.
Bold values indicate statistical significance ($P < 0.05$).

© 2025. Thomsen AK et al. Published online at http://www.aginganddisease.org/EN/10.14336/AD.2024.1585
